# From awareness to public health policy action: operational gaps in One Health implementation among frontline workers in Western India—A mixed method study

**DOI:** 10.3389/fpubh.2026.1814319

**Published:** 2026-04-23

**Authors:** Krishna Jasani, Krupal Joshi, Ashwini Agrawal, Om Prakash Bera, Margubur Rahaman, U. Venkatesh, Jayesh Vakani

**Affiliations:** 1 Community and Family Medicine Department, All India Institute of Medical Sciences, Rajkot, Gujarat, India; 2 Microbiology and Infectious Diseases Department, All India Institute of Medical Sciences, Rajkot, Gujarat, India; 3 Global Health Advocacy Incubator (GHAI), Washington, DC, United States; 4 All India Institute of Medical Sciences (AIIMS), Gorakhpur, India; 5 Community and Family Medicine Department, All India Institute of Medical Sciences, Gorakhpur, India; 6 Rajkot Municipal Corporation, Rajkot, Gujarat, India

**Keywords:** attitudes, front-line workers, knowledge, One Health, practices

## Abstract

**Background:**

India has increasingly endorsed One Health at the policy level. However, evidence on its operationalization at the district level, particularly among frontline public-sector workers, remains limited. This study assessed the awareness, knowledge, attitudes, and practices (AKAP) related to One Health among frontline personnel in Gujarat, India.

**Methods:**

A convergent mixed-methods study was conducted from January to June 2025 in Rajkot District, Gujarat. Quantitatively, 400 frontline workers from five departments (Health, AYUSH, Animal Husbandry, Food Safety, and Environment) were selected using proportionate stratified random sampling and interviewed using a validated AKAP questionnaire. Qualitatively, in-depth interviews were conducted with 25 purposively selected participants. Descriptive statistics, multivariable logistic regression, and exploratory factor analysis were performed for quantitative data, while thematic analysis was used for qualitative findings.

**Results:**

Overall, 72% of participants were aware of the One Health concept, and 71% demonstrated positive attitudes toward its implementation. However, only 45% reported good One Health practices. Knowledge and attitude scores were significantly higher among Health and Animal Husbandry personnel compared with Food Safety and Environment sectors (*p* < 0.001). Multivariable analysis identified high knowledge (aOR 2.8; 95% CI 1.9–4.2), positive attitude (aOR 2.3; 95% CI 1.6–3.4), prior One Health-specific training (aOR 3.1; 95% CI 2.0–4.9), and work experience >5 years (aOR 1.4; 95% CI 1.0–2.0) as independent predictors of good practice. Qualitative findings highlighted fragmented governance, limited interdepartmental coordination, lack of formal mandates, and absence of dedicated resources as major barriers to effective One Health implementation.

**Conclusions:**

Despite high awareness and favorable attitudes, the translation of One Health principles into routine practice remains limited at the district level. Institutionalizing structured training, strengthening intersectoral governance, and prioritizing inclusion of environmental and food safety sectors are essential to operationalize One Health effectively in India and similar low- and middle-income settings.

## Introduction

The concept of One Health has evolved over the past two centuries, originating as One *Medicine* and later progressing through concepts such as One *World, One Health* to the now widely recognized One *Health* framework (1). Although there is no universally accepted definition, several interpretations have been proposed. The most widely cited, shared by the U.S. Centers for Disease Control and Prevention (CDC) and the One Health Commission, defines One Health as “a collaborative, multisectoral, and transdisciplinary approach—working at the local, regional, national, and global levels—with the goal of achieving optimal health outcomes recognizing the interconnection between people, animals, plants, and their shared environment” (2). A central tenet of the One Health approach is to foster active collaboration and communication among diverse stakeholders across multiple sectors—not only at global and national levels but also at the community and local levels, where the interdependence between human, animal, and environmental health is most evident. This is particularly relevant to India, where a substantial proportion of the population resides in rural areas and engages in daily interactions with livestock and wildlife (3). The significance of the One Health approach has been reaffirmed by recent public health events in India. The recurring outbreaks of Nipah virus in Kerala, most recently in 2023, underscore how zoonotic viruses originating from fruit bats can cross species barriers and cause localized health emergencies requiring multisectoral coordination across human, veterinary, and environmental domains. Similarly, the rising incidence of scrub typhus in the Himalayan regions—including Himachal Pradesh and Uttarakhand—illustrates the impact of environmental change and human encroachment on forest ecosystems, which disrupt ecological balance and facilitate the spread of vector-borne zoonotic diseases (3). These examples highlight the urgent need for integrated surveillance systems, joint risk assessments, and community-level capacity building under the One Health framework. Within this context, Gujarat represents a compelling case for the operationalization of the One Health approach due to its large livestock population, agricultural base, and rapidly expanding industrial sectors. At the forefront of One Health implementation are the frontline workers—personnel from government and municipal departments such as Health, AYUSH, Animal Husbandry, Food Safety, and Environment. These individuals are responsible for monitoring, reporting, and responding to public health issues. Their effectiveness in implementing One Health principles depends largely on their awareness, knowledge, attitudes, and practices (AKAP) related to integrated health action (4). As the first responders during health crises, including zoonotic outbreaks, they play a vital role in surveillance, early detection, and containment of cross-species infections.

Despite increasing global recognition of the One Health approach, existing studies reveal limited awareness and understanding among frontline workers, particularly in rural and semi-urban settings (5). Although the approach has gained traction at the policy level in India, its translation into practice remains inconsistent due to gaps in interdepartmental coordination, resource constraints, and inadequate training in One Health concepts (3). This study therefore aims to assess the awareness, knowledge, attitudes, and practices of frontline workers toward the One Health approach, thereby identifying existing gaps and opportunities to strengthen its implementation at the grassroots level. In this study, “operationalization of One Health” refers to the practical implementation of intersectoral collaboration among human, animal, and environmental health systems through coordinated activities such as joint surveillance, information sharing, outbreak response, and preventive interventions at the field level.

### Objectives

- To evaluate the level of awareness and knowledge of frontline workers regarding the principles and importance of the One Health approach.
- To analyse the attitudes of frontline workers toward the implementation and benefits of the One Health in their respective sectors.
- To examine the current practices adopted by frontline workers in relation to the One Health approach.
- To identify gaps and challenges in the awareness, knowledge, attitudes, and practices of frontline workers that may affect the effective implementation of the One Health approach at the district level.

## Materials and methods

### Study design and setting

This was a convergent mixed-methods study (quantitative and qualitative) conducted from January to June 2025 in Rajkot District, Gujarat, a region with diverse ecological and occupational exposure patterns relevant to the One Health (OH) framework. The study aimed to evaluate awareness, knowledge, attitudes, and practices (AKAP) related to One Health among front-line personnel. The research design was informed by the *Tripartite One Health Joint Plan of Action* and complied with the STROBE and *COREQ* guidelines for observational and qualitative research, respectively.

### Study population

The study targeted front-line public-sector workers actively engaged in disease surveillance, animal health, food safety, or environmental regulation. Five government departments were selected purposively: Health, AYUSH (Ayurveda, Yoga, Unani, Siddha, and Homoeopathy), Animal Husbandry and Veterinary Services, Food Safety, and Environment and Pollution Control. These departments represent key sectors in operationalizing One Health strategies at the district level.

### Inclusion and exclusion criteria

Eligible participants were those aged 18 years or older, with a minimum of six months' service in their respective departments, and involved in fieldwork or supervisory activities. All participants were required to provide written informed consent. Exclusion criteria included being on extended leave during the study period, inability to respond to an interviewer-administered questionnaire due to language or cognitive limitations, and refusal to participate.

### Sampling frame and sample size

The sampling frame was derived from official departmental human resource rosters updated in December 2024, identifying 1,340 eligible front-line personnel across the five sectors. A proportionate stratified random sampling technique was employed to ensure equitable representation across departments. The minimum required sample size was calculated using the single population proportion formula, with a 40% prevalence of good OH practice, 5% absolute precision, 95% confidence level, and a design effect of 1.2. This yielded a target of 384 participants, which was inflated to 400 to account for a potential 5% non-response rate. Stratified allocations were made based on department-specific workforce proportions.

For the qualitative component, a subset of 25 participants was randomly selected across departments and experience levels to ensure diversity. Data saturation was used as the guiding principle for sample adequacy.

### Data collection tool

#### Quantitative tool

A structured questionnaire was developed after an extensive literature review and Delphi consultation with ten OH experts. It included six sections:

- Sociodemographic details
- Awareness of the OH concept
- 12-item multiple-choice knowledge scale (maximum score = 10)
- 15-item Likert-type attitude scale (scored 1–5)
- 6-item practice scale (score range: 0–6)
- Open- and close-ended questions on perceived barriers/enablers

The questionnaire was developed in English, translated into Gujarati, and back-translated to ensure linguistic accuracy. Pretesting among 20 non-study participants confirmed clarity and usability. The tool showed high reliability (CVI = 0.96; Cronbach's α = 0.88 for knowledge and 0.91 for attitude).

#### Qualitative tool

A semi-structured interview guide was used to elicit participants' experiences, perceptions, and recommendations regarding One Health implementation. Key domains included:

- Perceived intersectoral collaboration
- Communication and coordination barriers
- Institutional enablers and leadership roles
- Field-level challenges in zoonotic disease prevention and environmental surveillance

Interviews (30–45 min each) were conducted in Gujarati or English by trained researchers, audio-recorded with permission, and transcribed verbatim.

### Study variables and definitions

The 6-item practice scale was developed based on an extensive review of existing One Health operational frameworks, including intersectoral collaboration, zoonotic disease surveillance, outbreak preparedness, and environmental health coordination. Items were generated to reflect routine field-level practices relevant to frontline workers across sectors. Content validity was ensured through Delphi consultation with ten One Health experts, and each item was structured as a binary or frequency-based response, yielding a total score ranging from 0 to 6, with higher scores indicating better practice. The primary outcome was good OH practice, defined as a composite practice score of ≥4 out of 6. Key independent variables included high knowledge (score ≥ 7/10), positive attitude (average score ≥ 4/5), and prior participation in OH-specific training (defined as any formal training lasting ≥1 day in the past 3 years). Additional covariates included department, sex, age group, education level, and years of professional experience. These were treated as categorical variables during analysis. The practice-related items were included in the pretesting phase conducted among 20 non-study participants to assess clarity, feasibility, and contextual relevance. Minor modifications in wording were made based on participant feedback to improve comprehension (Table 1).

**Table 1:** Operational definitions of outcome, exposure variables, and covariates.

Domain	Variable(s)	Operational definition
Outcome	Good practice	Composite practice score ≥ 4 of 6
Primary exposures	High knowledge; Positive attitude; OH-specific training	Knowledge score ≥ 7/10; Attitude index ≥ 4/5; Any formal OH course ≥ 1 day in past 3 years
Covariates	Department; sex; age group; education level; years of experience	Categorical as specified in Results

### Data collection procedure

Data were collected by trained investigators who conducted interviewer-administered surveys in private areas during working hours to minimize reporting bias. Investigators were blinded to the specific study hypotheses. Completed questionnaires were reviewed for completeness and consistency and double-entered into a secure REDCap database, with in-built logic checks and range validations.

For qualitative data, in-depth interviews were conducted face-to-face at participants' workplaces or neutral venues. Field notes and reflective memos were maintained to support contextual interpretation.

### Statistical and thematic analysis

All statistical analyses were performed using Jamovi version 7.0 Descriptive statistics were used to summarize participant characteristics. Continuous variables were presented as means with standard deviations or medians with interquartile ranges, depending on normality. Categorical variables were summarized as frequencies and percentages. Reliability of the knowledge and attitude scales was assessed using Cronbach's alpha. Exploratory factor analysis with principal axis factoring and varimax rotation was conducted to identify latent constructs within the attitude scale (KMO test and Bartlett's test used for suitability assessment). Group comparisons of knowledge and attitude scores across departments were analyzed using ANOVA with *post-hoc* Tukey tests, or Kruskal-Wallis and Mann–Whitney *U*-tests where assumptions of normality were not met. Chi-square or Fisher's exact tests were used to assess associations between categorical variables. Correlations among KAP scores were assessed using Pearson's or Spearman's coefficients.

A binary logistic regression model was constructed to identify independent predictors of good OH practice. Variables with *p* < 0.20 in univariate analysis were included in the multivariable model. Adjusted odds ratios (aORs) with 95% confidence intervals were reported. Model fit was evaluated using the Hosmer–Lemeshow test, and predictive accuracy using the area under the receiver operating characteristic curve (AUC). Multicollinearity was checked using variance inflation factors (VIFs). Radar plots were used to visualize normalized departmental KAP profiles, and a forest plot was created to display adjusted odds ratios with 95% CI.

#### Qualitative analysis

Qualitative data were analyzed using thematic analysis following Braun and Clarke's six-step approach:

1. Familiarization with transcripts
2. Initial coding
3. Searching for themes
4. Reviewing themes
5. Defining and naming themes
6. Producing the report

Coding was performed manually by two independent researchers and validated through peer debriefing to ensure consistency. Themes were triangulated with quantitative results to provide a comprehensive understanding of OH operationalization barriers and enablers.

Integration of qualitative and quantitative components was performed using a convergent mixed-methods approach, wherein both datasets were collected and analyzed concurrently. Findings were integrated at the interpretation and reporting stage by embedding qualitative insights within corresponding quantitative domains (awareness, knowledge, attitude, and practice) to enable triangulation and enhance contextual understanding.

### Ethical considerations

The study was reviewed and approved by the Institutional Ethics Committee of AIIMS Rajkot (Approval number: IEC/AIIMS/RKT/2024-073, dated 22 December 2024). All participants provided written informed consent. Data were anonymised and stored in password-protected systems accessible only to the core research team. The study complied with ethical principles outlined in the Declaration of Helsinki (2013 revision).

## Results

### Sociodemographic characteristics

A total of 400 front-line personnel from five key public sector departments were included in the analysis. These comprised workers from the Health Department [150 (37.5%)], AYUSH [80 (20.0%)], Animal Husbandry [80 (20.0%)], Food Safety [45 (11.3%)], and the Environment Department [45 (11.3%)]. The median age of participants was 36 years [interquartile range (IQR) 29–42], and the gender distribution skewed slightly male (57%). Most respondents (67%) held a graduate-level degree or higher qualification. Forty-one percent of participants reported having five or more years of work experience in their respective departments. Departmental distribution, age, gender, education, and experience levels did not significantly differ with regard to baseline awareness of the One Health approach (Table 2).

**Table 2:** Sociodemographic characteristics of participants involved in the study.

Variable	Category	*n* (%)
Departments involved	Health	150 (37.5)
	AYUSH	80 (20.0)
	Animal Husbandry	80 (20.0)
	Food Safety	45 (11.3)
	Environment	45 (11.3)
Age group (years)	<30	92 (23.0)
	30–39	166 (41.5)
	40–49	98 (24.5)
	≥50	44 (11.0)
Gender	Male	228 (57.0)
	Female	172 (43.0)
Education level	Graduate or higher	268 (67.0)
	Below graduate	132 (33.0)
Work experience	<5 years	236 (59.0)
	≥5 years	164 (41.0)
Awareness of One Health	Yes	288 (72.0)
	No	112 (28.0)
Awareness by department	Health	125/150 (83.3)
	Animal Husbandry	61/80 (76.3)
	AYUSH	57/80 (71.3)
	Food Safety	30/45 (66.7)
	Environment	26/45 (57.8)
Sources of awareness (among those aware, *n* = 288)	Workplace-based formal training	132 (45.8)
	Conferences/seminars	110 (38.2)
	Academic curricula	81 (28.1)
	Digital media platforms	61 (21.2)

Interviews revealed that respondents across departments viewed their professional background and field experience as critical in shaping their understanding of intersectoral health linkages. However, several participants, particularly from the AYUSH and Environment sectors, expressed that “*roles within the One Health framework are still unclear at the field level.”* Participants also highlighted limited opportunities for cross-sectoral exposure during their early training or service postings.

### Awareness of the One Health approach

Overall, 288 of 400 participants (72%) reported having heard of the “One Health” concept prior to the survey. Awareness was highest among respondents from the Health Department (83%), followed by Animal Husbandry (76%), AYUSH (71%), Food Safety (66%), and lowest in the Environment Department (58%). The association between departmental affiliation and awareness levels was statistically significant (χ^2^ = 15.8; df = 4; *p* = 0.003). Among those aware of One Health, the most frequently cited sources of information were workplace-based formal training (46%) and professional conferences or seminars (38%). Fewer participants reported acquiring awareness via academic curricula (28%) or digital media platforms (21%), suggesting a predominance of institution-driven exposure.

**Figure 1:**
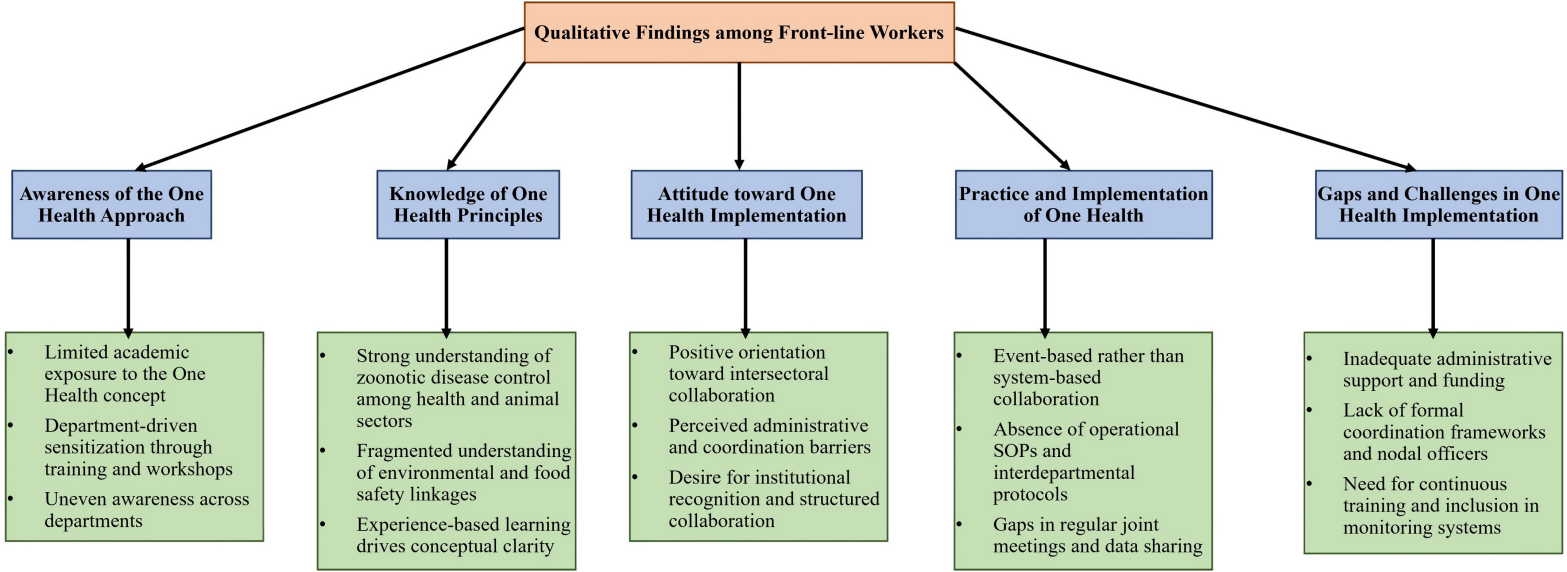
Thematic analysis of qualitative findings among front-line workers on awareness, knowledge, attitudes, and practices toward the one health approach.

Qualitative analysis revealed that; Participants commonly described the One Health approach as “a shared responsibility across departments,” though many admitted to having only a “surface-level understanding.” Respondents emphasized that awareness often depended on “*whether the department had organized workshops or cross-sectoral meetings.”* Several frontline workers noted that One Health had not been included in their initial training curriculum, highlighting an academic and institutional gap in systematic sensitization (Figure 1).

### Knowledge scores

The mean composite knowledge score on a 10-point scale was 7.0 ± 1.9. A score of ≥7, indicating high knowledge, was observed in 248 participants (62%). Mean scores were highest in the Health (7.6 ± 1.7) and Animal Husbandry (7.2 ± 1.8) sectors, followed by AYUSH (6.9 ± 1.6), Food Safety (6.4 ± 1.5), and Environment (6.1 ± 1.4). One-way analysis of variance (ANOVA) revealed statistically significant differences in mean knowledge scores across departments (*F* = 6.4; *p* < 0.001). *Post-hoc* pairwise comparisons using Tukey's HSD test demonstrated that the Food Safety and Environment departments scored significantly lower than the Health department (*p* < 0.01 in both comparisons), while the Animal Husbandry and AYUSH departments did not show statistically significant differences. The internal consistency of the 12-item knowledge scale was high (Cronbach's α = 0.88). Pearson's correlation analysis identified a modest but statistically significant positive association between years of experience and knowledge score (*r* = 0.28; *p* < 0.001), suggesting that prolonged field engagement may contribute to enhanced conceptual understanding of One Health.

Supporting qualitative insights revealed that; Respondents with higher knowledge often attributed it to their “*involvement in outbreak control or zoonotic surveillance activities.”* Others noted that while they were aware of zoonotic disease concepts, “*integration with environmental or food safety aspects was rarely discussed.”* Environmental officers particularly expressed a lack of structured technical modules to understand the “health-environment-animal interface” (Figure 1).

### Attitudinal disposition toward One Health

Attitudes toward One Health were measured using a validated 15-item Likert scale. The overall median attitude score (range: 1–5) was 4.1 (IQR 3.7–4.5), and 284 participants (71%) expressed a positive or strongly positive orientation toward adopting the One Health model. Exploratory factor analysis using principal axis factoring with varimax rotation yielded a three-factor solution explaining 68% of the total variance. These factors were labeled as: (i) *Perceived Importance of One Health*, (ii) *Commitment to Intersectoral Collaboration*, and (iii) *Resource Adequacy and Administrative Support*. Sampling adequacy was confirmed by a high KMO statistic (0.91), and Bartlett's test of sphericity was significant (*p* < 0.001), supporting data suitability. Prior One Health-specific training was significantly associated with higher attitudinal scores (Mann–Whitney *U* = 11,122; *p* < 0.001). Additionally, a positive correlation was observed between knowledge and attitude scores (*r* = 0.35; *p* < 0.001), indicating a knowledge-driven attitudinal shift.

Narrative findings further indicated broad enthusiasm for the One Health approach, with several respondents stating that “*joint efforts could make disease prevention more efficient.”* However, many expressed frustration over the lack of interdepartmental communication and overlapping jurisdictional boundaries. Some participants felt “*motivation is high, but administrative support and coordination are weak.”* A few highlighted the absence of performance indicators or recognition for One Health-related initiatives in their departments (Figure 1).

### Practice patterns and implementation behaviors

Good practice was defined as a composite score ≥ 4 on a 6-point scale measuring engagement in One Health-related activities such as interdepartmental collaboration, outbreak preparedness, and zoonotic surveillance. Overall, only 45% (*n* = 180) of participants met this threshold. Intersectoral meetings were infrequent, with only 32% reporting quarterly or more frequent joint meetings. The most commonly cited operational barriers were “limited financial and infrastructural resources” (54%), “poor coordination across departments” (48%), and “lack of clear guidelines or mandates” (38%). Notably, only 28% of participants reported access to formal SOPs or documentation outlining One Health roles in their institutions.

Qualitative responses highlighted that; Frontline workers consistently mentioned “*reactive collaboration during outbreaks”* rather than proactive intersectoral engagement. Many described One Health implementation as “*event-driven rather than system-driven.”* Participants emphasized that despite willingness to collaborate, “*each department operates in silos,”* citing bureaucratic delays and lack of clarity on leadership roles. Suggestions included periodic joint meetings, designated nodal officers, and integrating One Health indicators into departmental monitoring frameworks.

### Determinants of good One Health practice

Multivariable binary logistic regression identified predictors of good practice. The model demonstrated good fit (Hosmer–Lemeshow *p* = 0.47; AUC = 0.79). After adjustment for age, gender, and experience, the following factors were independently associated with good practice:

- High knowledge score (aOR 2.8; 95% CI 1.9–4.2; *p* < 0.001)
- Positive attitude (aOR 2.3; 95% CI 1.6–3.4; *p* < 0.001)
- Prior One Health-specific training (aOR 3.1; 95% CI 2.0–4.9; *p* < 0.001)
- Work experience > 5 years (aOR 1.4; 95% CI 1.0–2.0; *p* = 0.047)

**Figure 2:**
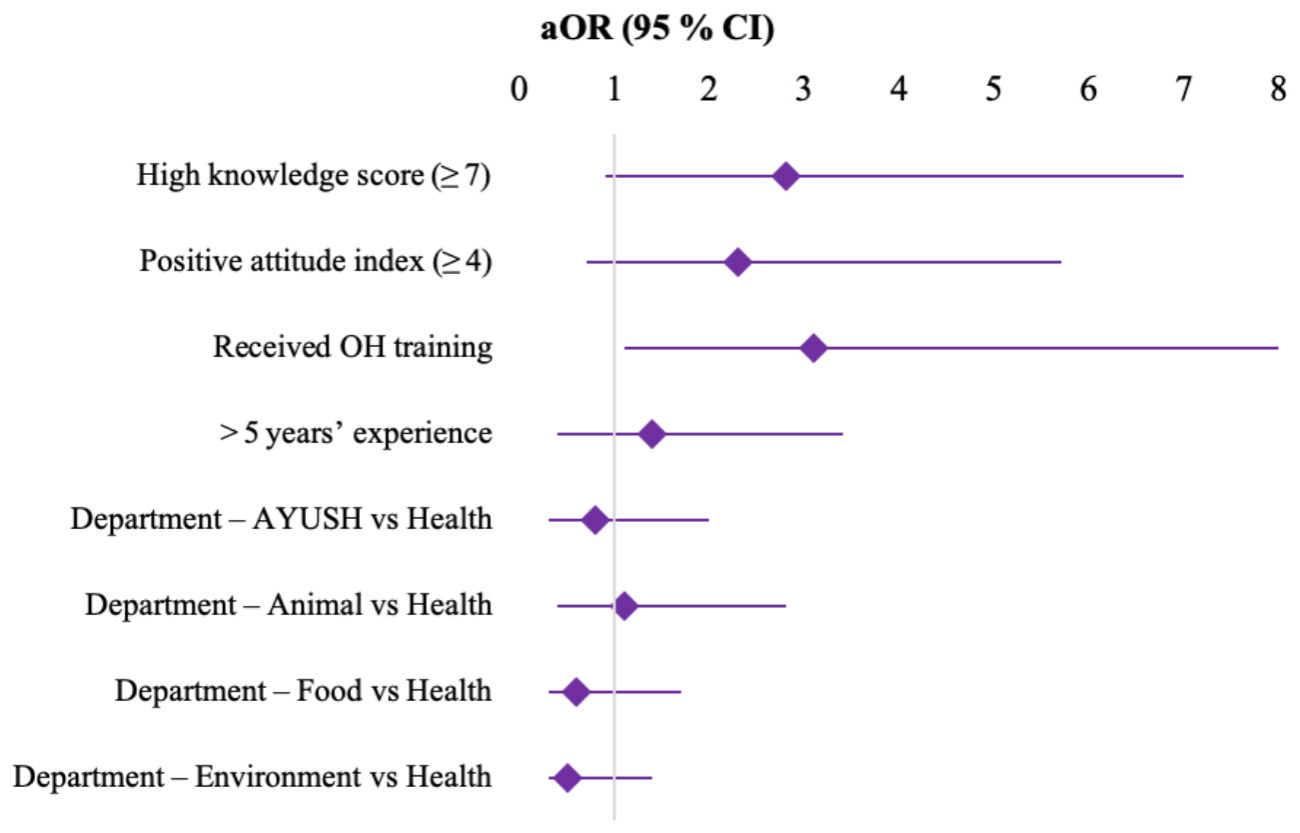
Forest plot showing adjusted odds ratios (aOR) with 95% confidence intervals for predictors of good One Health practices among frontline workers. Predictors included high knowledge score (≥7), positive attitude index (≥4), receipt of One Health (OH) training, work experience >5 years, and departmental affiliation (AYUSH, Animal Health, Food Safety, and Environment) compared with the Health Department as the reference category. The vertical line at aOR = 1 represents no association.

Compared to the Health Department, respondents from the Environment Department were significantly less likely to exhibit good practice behaviors (aOR 0.5; 95% CI 0.2–0.9; *p* = 0.02). No statistically significant differences were observed among AYUSH, Animal Husbandry, and Food Safety sectors relative to Health (Table 3; Figure 2).

**Table 3:** Multivariable logistic regression analysis showing predictors of good one health practices among frontline workers.

Predictor	aOR (95 % CI)	*p*-value
High knowledge score (≥ 7)	2.8 (1.9–4.2)	<0.001
Positive attitude index (≥ 4)	2.3 (1.6–3.4)	<0.001
Received OH training	3.1 (2.0–4.9)	<0.001
>5 years' experience	1.4 (1.0–2.0)	0.047
Department – AYUSH vs. Health	0.8 (0.5–1.2)	0.25
Department – Animal vs. Health	1.1 (0.7–1.7)	0.68
Department – Food vs. Health	0.6 (0.3–1.1)	0.09
Department – Environment vs. Health	0.5 (0.2–0.9)	0.02

Supporting qualitative insights revealed that; Participants frequently identifies administrative inertia, fragmented data-sharing mechanisms, and lack of dedicated budget lines as critical barriers. One senior health officer remarked that “*collaboration ends once the outbreak is contained.”* Several interviewees recommended establishing “*district-level One Health coordination cells”* to sustain collaboration and policy alignment (Figure 1).

### Interdepartmental comparison of KAP scores

**Figure 3:**
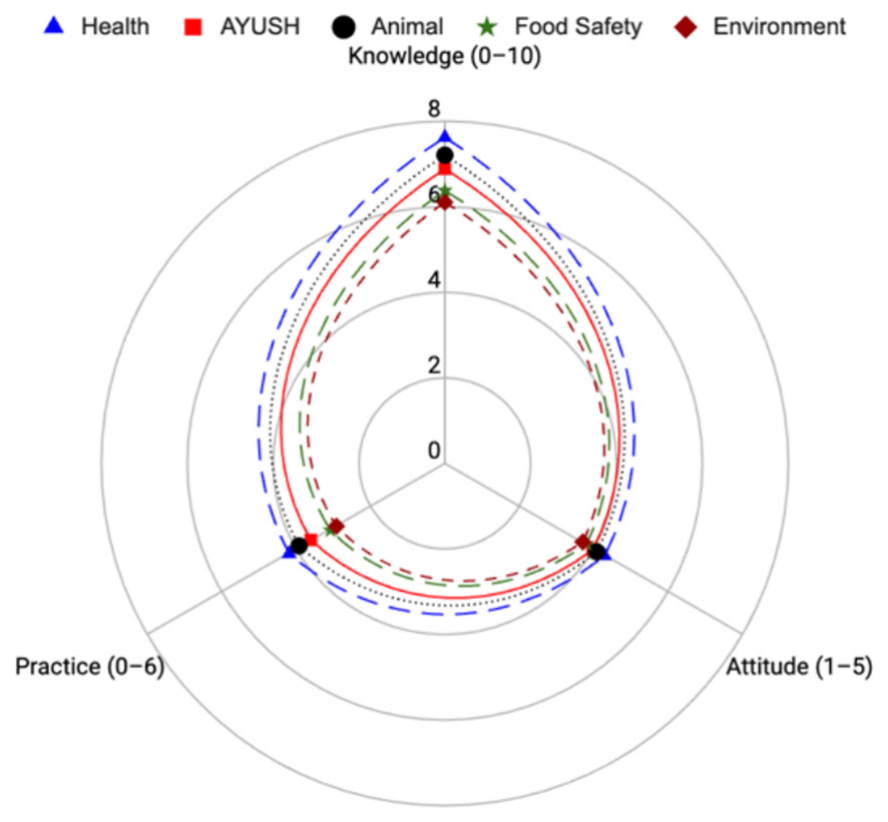
Radar plot comparing Knowledge, Attitude, and Practice (KAP) scores across different departments involved in the study. Radar plot comparing mean Knowledge (0–10), Attitude (1–5), and Practice (0–6) scores among frontline workers across departments, including Health, AYUSH, Animal Health, Food Safety, and Environment. Each axis represents one KAP domain, and plotted values indicate the average scores for each department.

To better visualize interdepartmental variation, a radar plot was generated using normalized average Knowledge, Attitude, and Practice (KAP) scores for each department. The radar plot (Figure 3) revealed that the Health and Animal Husbandry departments consistently scored highest across all KAP domains, whereas the Food Safety and Environment departments exhibited the lowest performance. This visual stratification highlights critical gaps in workforce readiness and underscores the need for targeted capacity-building.

## Discussion

This mixed-methods study provides district-level evidence on the awareness, knowledge, attitudes, and practices (AKAP) related to the One Health (OH) approach among frontline public-sector workers in India. While awareness and attitudinal acceptance of One Health were relatively high, the translation of these constructs into routine practice remained limited. These findings highlight a persistent gap between conceptual endorsement and operational implementation of One Health at the grassroots level.

In the present study, nearly three-quarters of frontline workers reported prior awareness of the One Health concept, with significantly higher awareness among Health and Animal Husbandry personnel compared with Food Safety and Environment departments. This sectoral gradient mirrors findings from both Indian and international studies, where One Health awareness is often driven by engagement in zoonotic disease control and outbreak response rather than through systematic education or cross-sectoral training (6–8). Evidence from India suggests that exposure to One Health concepts largely occurs through *ad hoc* workshops and disease-specific programs, rather than through formal academic curricula or induction training (8, 9).

Knowledge scores followed a similar pattern, with Health and Animal Husbandry departments demonstrating significantly higher mean scores than Food Safety and Environment sectors. Comparable findings have been reported from Ethiopia, Bangladesh, and Southeast Asia, where frontline workers in human and veterinary health systems show stronger understanding of zoonotic linkages, while environmental and food safety professionals report fragmented or incomplete conceptualization of One Health (6, 10–12). The relatively lower scores observed among the Environment and Food Safety sectors may be attributed to their comparatively limited integration within routine health surveillance systems and fewer opportunities for intersectoral engagement. Unlike Health and Animal Husbandry departments, which are directly involved in outbreak detection and response, these sectors often operate within regulatory or monitoring frameworks with less direct participation in coordinated field-level activities. Additionally, the absence of clearly defined roles, limited access to One Health-specific training, and weaker institutional linkages with health-led programs may further constrain their translation of knowledge into practice. The positive association between years of professional experience and knowledge observed in this study suggests that experiential learning, particularly during outbreak investigations, compensates partially for the lack of structured training.

Qualitative findings further reinforced this interpretation, as respondents frequently attributed their knowledge to participation in surveillance or outbreak control activities. However, many participants acknowledged that integration of environmental and food system perspectives into routine public health practice remains weak. This fragmented understanding has been consistently identified as a major barrier to effective One Health implementation in low- and middle-income countries (LMICs) (7, 11, 13).

Attitudinal assessment revealed broad support for the One Health approach, with more than 70% of participants expressing positive or strongly positive attitudes. This finding aligns with international literature demonstrating that frontline professionals generally recognize the value of intersectoral collaboration, even in settings where operational frameworks are underdeveloped (9, 13, 14). Studies from South Asia similarly report high normative acceptance of One Health principles among government personnel, despite limited institutional mechanisms to support collaboration (3, 15). The observed positive correlation between knowledge and attitudes supports behavioral theory suggesting that improved understanding fosters favorable dispositions toward collective action (16). Nevertheless, qualitative narratives highlighted frustration related to administrative inertia, unclear leadership, and lack of incentives or recognition for One Health-related activities. These barriers have been widely documented across LMIC contexts, where siloed governance structures and overlapping mandates undermine sustained intersectoral engagement (3, 14, 15).

Exploratory factor analysis identified “resource adequacy and administrative support” as a key attitudinal domain, underscoring the importance of institutional enablers in shaping frontline perceptions. Similar findings have been reported in global One Health evaluations, which emphasize that positive attitudes alone are insufficient to drive practice without supportive governance, financing, and accountability frameworks (3, 13, 17).

Despite relatively high awareness and favorable attitudes, fewer than half of participants demonstrated good One Health practices, revealing a pronounced knowledge–practice gap. This pattern is consistent with findings from multiple international studies, where One Health implementation remains largely reactive and crisis-driven rather than institutionalized within routine systems (3, 10, 18). In the present study, intersectoral collaboration was most commonly reported during outbreaks, with limited evidence of sustained joint planning, data sharing, or preventive action. Similar event-based collaboration has been described during zoonotic disease responses in India and other LMICs, where coordination intensifies during emergencies but rapidly declines once the immediate threat subsides (18, 19). The low availability of formal SOPs, infrequent joint meetings, and lack of designated coordination structures observed in this study reflect systemic challenges in embedding One Health into district-level governance.

Qualitative findings provided critical contextual insights, with frontline workers describing One Health implementation as “event-driven rather than system-driven.” Participants consistently emphasized the absence of clear mandates, nodal leadership, and dedicated budgetary provisions—barriers that have also been highlighted in regional and global One Health assessments (13, 17).

Multivariable analysis identified high knowledge, positive attitudes, prior One Health-specific training, and longer work experience as independent predictors of good practice. These findings are consistent with evidence from Ethiopia, and Rwanda where formal training exposure emerged as one of the strongest determinants of effective intersectoral engagement (6, 20). The magnitude of association between training and good practice observed in this study underscores the critical role of structured capacity-building initiatives. Notably, frontline workers from the Environment Department were significantly less likely to demonstrate good One Health practices compared with their Health counterparts, even after adjustment. This highlights a critical equity gap in One Health preparedness across sectors. Environmental professionals are often peripheral to health-led surveillance systems, despite their central role in addressing ecological drivers of zoonotic risk, climate change, and food system vulnerabilities (17, 21). Strengthening the integration of environmental health within One Health governance is therefore essential for comprehensive risk mitigation. Triangulation of quantitative and qualitative findings strengthens the validity of this study. While survey data quantified gaps in practice, qualitative narratives elucidated underlying governance, coordination, and resource constraints. The repeated recommendation for district-level One Health coordination cells aligns with international guidance advocating decentralized, context-specific governance mechanisms to sustain multisectoral collaboration (14, 22).

This study is among the few in India to employ a convergent mixed-methods design to assess One Health operationalization at the district level across multiple departments. The use of validated tools, robust sampling, and methodological triangulation enhances credibility. However, the cross-sectional design limits causal inference, and self-reported practices may be subject to social desirability bias. Additionally, findings from a single district may limit generalizability, although they provide valuable insights for similar settings. The findings of this study have important implications for India's evolving One Health agenda. First, One Health concepts must be systematically integrated into pre-service education and in-service training across all relevant sectors. Second, institutional mechanisms—including designated nodal officers, routine joint reviews, shared surveillance platforms, and dedicated funding—are essential to translate awareness and positive attitudes into sustained practice. Third, targeted capacity-building for environmental and food safety sectors is necessary to ensure balanced and inclusive One Health implementation.

## Conclusion

In conclusion, frontline workers in Rajkot District demonstrate substantial awareness and favorable attitudes toward the One Health approach; however, effective implementation remains constrained by structural, administrative, and coordination-related barriers. Bridging this gap requires moving beyond policy endorsement toward institutionalization of training, governance, and accountability mechanisms. Strengthening frontline capacity and intersectoral systems is essential to realize the full potential of One Health in addressing complex health threats in India and comparable LMIC settings. At the district level, establishing dedicated One Health coordination cells with representation from all relevant departments can facilitate routine intersectoral communication and joint planning. Additionally, implementing periodic joint training programs and simulation exercises can strengthen collaborative competencies and improve preparedness for zoonotic and environmental health threats.

## Data Availability

The original contributions presented in the study are included in the article/supplementary material, further inquiries can be directed to the corresponding author.
